# Qingda granule exerts neuroprotective effects against ischemia/reperfusion-induced cerebral injury via lncRNA GAS5/miR-137 signaling pathway

**DOI:** 10.7150/ijms.53603

**Published:** 2021-02-06

**Authors:** Ling Zhang, Qiaoyan Cai, Shan Lin, Bin Chen, Beibei Jia, Renzhi Ye, Nathaniel Weygant, Jianfeng Chu, Jun Peng

**Affiliations:** 1 Academy of Integrative Medicine, Fujian University of Traditional Chinese Medicine, 1 Qiuyang Road, Minhou Shangjie, Fuzhou 350122, China.; 2 Fujian Key Laboratory of Rehabilitation Technology, Fujian University of Traditional Chinese Medicine, Qiuyang Road, Minhou Shangjie, Fuzhou, China.; 3 Fujian Key Laboratory of Integrative Medicine on Geriatrics, Fujian University of Traditional Chinese Medicine, 1 Qiuyang Road, Minhou Shangjie, Fuzhou 350122, China.; 4 Chen Keji Academic Thought Inheritance Studio, Fujian University of Traditional Chinese Medicine, 1 Qiuyang Road, Minhou Shangjie, Fuzhou, Fujian 350122, China.; 5 People's Hospital of Fujian University of Traditional Chinese Medicine, No.602, 817 Middle Road, Fuzhou 350004, China.; 6 The Higher Educational Key Laboratory for Integrative Medicine of Fujian Province, Fujian University of Traditional Chinese Medicine, 1 Qiuyang Road, Minhou Shangjie, Fuzhou, Fujian 350122, China.

**Keywords:** Qingda granule, ischemic stroke, lncRNA GAS5, miR-137, neuronal apoptosis

## Abstract

**Background:** Ischemic stroke is the second leading cause of death and disability worldwide, which needs to develop new pharmaceuticals for its prevention and treatment. Qingda granule (QDG), a traditional Chinese medicine formulation, could improve angiotensin II-induced brain injury and decrease systemic inflammation. In this study, we aimed to evaluate the neuroprotective effect of QDG against ischemia/reperfusion-induced cerebral injury and illustrate the potential mechanisms.

**Methods:** The middle cerebral artery occlusion/reperfusion (MCAO/R) surgery *in vivo* and oxygen-glucose deprivation/reoxygenation (OGD/R) *in vitro* models were established. Ischemic infarct volume was quantified using magnetic resonance imaging (MRI). Neurobehavioral deficits were assessed using a five-point scale. Cerebral histopathology was determined by hematoxylin-eosin (HE) staining. Neuronal apoptosis was evaluated by TUNEL and immunostaining with NeuN antibodies. The protective effect of QDG on OGD/R-injured HT22 cells was determined by MTT assay and Hoechst 33258 staining. The expression of lncRNA GAS5, miR-137 and apoptosis-related proteins were investigated in MCAO/R-injured rats and in OGD/R-injured HT22 cells using RT-qPCR and western blot analysis.

**Results:** QDG significantly reduced the ischemic infarct volume, which was accompanied with improvements in neurobehavioral deficits. Additionally, QDG significantly ameliorated cerebral histopathological changes and reduced neuron loss in MCAO/R-injured rats. Moreover, QDG improved growth and inhibited apoptosis of HT22 cells injured by OGD/R *in vitro*. Finally, QDG significantly decreased the expression of lncRNA GAS5, Bax and cleaved caspase3, whereas it increased miR-137 and Bcl-2 expression in MCAO/R-injured rats and in OGD/R-injured HT22 cells.

**Conclusion:** QDG plays a neuroprotective role in ischemic stroke via regulation of the lncRNA GAS5/miR-137 signaling pathway.

## Introduction

Stroke is the second most frequent cause of death and a leading cause of long-term severe disability worldwide 1. Strokes are classified as either ischemic or hemorrhagic, and approximately 85% of strokes are ischemic 2. When an embolus or thrombus blocks the blood supply, the affected part of the brain is unable to function properly, which is accompanied with brain cell death leading to severe disability or death. Currently, the clinical therapeutic intervention for acute ischemic stroke is thrombolysis such as intravenous injection of recombinant tissue plasminogen activator (tPA). However, therapeutic efficacy is limited by a narrow window of time for administration. Moreover, reperfusion after thrombolysis can exacerbate injury and greatly decrease therapeutic efficacy 3. Ischemic stroke is triggered by a combination of genetic and environmental factors. The main pathogenesis of ischemic stroke includes neurotoxicity of excitatory amino acids, calcium overload, blood-brain barrier damage, hypoxia, inflammatory reactions, oxidative stress and edema formation 4. These factors together induce neuronal apoptosis and necrosis, eventually leading to neurological dysfunction and death in the ischemic core. Nevertheless, studies have shown that neuronal apoptosis in the ischemic penumbra of cerebral infarction is reversible 5, 6. Therefore, there is an urgent need to explore the mechanisms of neuronal apoptosis and develop new therapies to alleviate brain damage following ischemic stroke.

Traditional Chinese medicines (TCMs) have been widely used in neurological therapy demonstrating remarkable improvements in neurological functions of patients with ischemia/reperfusion (I/R) diseases 7. Qingxuan Jiangya Decoction (QXJYD), a formulation prescribed by Chen Keji, has been used in clinic over 60 years for the treatment of hypertension (a high risk factor for stroke). Qingda granule (QDG) is derived from QXJYD, has been shown to alleviate brain injury and decrease systemic inflammation in angiotensin II-induced hypertensive mice 8. However, the therapeutic effect and potential mechanisms of QDG in ischemic stroke remain unknown.

It has been reported that cerebral ischemia is greatly impacted by the expression of long non-coding RNAs (lncRNAs) in the development of brain and neurodegenerative disorders, highlighting lncRNAs promising use as potential diagnostic biomarkers and therapeutic approaches for stroke 9-11. The lncRNA growth arrest‐specific transcript 5 (GAS5), firstly identified in NIH 3T3 mouse fibroblasts, is a crucial role for inhibiting cell survival in various cancers 12, 13. It has been reported that lncRNA GAS5 could drastically upregulate in the neuron under hypoxia and in the brain of rats subjected to transient focal ischemia 14, 15. Moreover, lncRNA GAS5 may also act as a competing endogenous RNA (ceRNA) to regulate specific RNA transcripts by competing with shared miRNAs 16, 17. Previous studies have indicated that lncRNA GAS5 acted as a ceRNA to decrease miR-137 expression, thus promoting the development of ischemic stroke 15. Bioactive compounds derived from Chinese herbs could target various lncRNAs to display their protective effects on stroke 18. However, whether QDG could govern cerebral I/R damage and post-ischemia recovery via these lncRNA‐miRNA crosstalk remains unclear. Therefore, the current study was carried out to assess the effects of QDG against neurological impairment in rats after cerebral I/R injury and explore the underlying mechanisms. The findings of this study may provide a theoretical basis for the clinical application of QDG as a novel therapy in the prevention and treatment for ischemic stroke.

## Methods

### Materials and reagents

Hematoxylin/eosin dye solution was purchased from China solarbio technology co., Ltd. Antigen retrieval solution and immunohistochemical staining kit were purchased from Fuzhou Maixin biotechnology development co., Ltd. TUNEL apoptosis detection kit and Hoechst 33258 kit were provided by KeyGen Biotech. Co. Ltd (Nanjing, Jiangsu, China). Bcl-2 and cleaved caspase3 antibodies were purchased from Cell Signaling Technology (Beverly, MA, USA). NeuN antibody was obtained from Abcam (Cambridge, MA, USA). Bax and β-actin antibodies were provided from Wuhan Sanying Biotechnology Co. Ltd (Hubei, Wuhan, China). TRIzol reagent, PrimeScript RT reagent kit and SYBR Premix Ex Taq Ⅱ kit were provided by Dalian Takara Biotechnology Co., Ltd. (Dalian, Liaoning, China). All of the other chemicals were obtained from Sigma-Aldrich (St. Louis, MO, USA).

### Preparation of QDG

QDG was prepared and provided by Jiangyin Tianjiang Pharmaceutical Co., Ltd. (Jiangsu, China; batch number: 1704306). QDG was dissolved in 0.9% NaCl for *in vivo* experiments, and in phosphate-buffered saline (PBS) to a final concentration of 5 mg/mL for *in vitro* experiments. Solutions were stored until use at -20 °C.

Baicalin was quantified to ensure the quality of the granule via using Ultra Performance Liquid Chromatography (UPLC). In the UPLC fingerprint profile, the peak of baicalin was identified by comparing the retention time of the corresponding baicalin standard, revealing the main component of QDG was baicalin. Please refer to the supplementary information for further details.

### Cell culture

Mouse hippocampal neuron HT22 cells were purchased from Shanghai Zeye Biotechnology Co., Ltd, and cultured in Dulbecco's Modified Eagle's medium (DMEM) with 10% fetal bovine serum, 100 U/mL penicillin and 100 µg/mL streptomycin at 37 °C in 5%° CO_2_ humidified air.

### *In vitro* model of oxygen and glucose deprivation/reoxygenation (OGD/R)

OGD/R was established to mimic ischemia and reperfusion *in vitro*. Briefly, the culture medium of HT22 cells was removed and replaced with glucose-free DMEM (Gibco, Thermo Fisher Scientific Inc., Waltham, MA, USA). Subsequently, the HT22 cells were cultured in a humidified chamber containing 1% O_2_, 5% CO_2_, and 94% N_2_ at 37 °C for 4 h. Thereafter, the HT22 cells were returned to normoxic conditions with normal medium for another 24 h for reoxygenation. Cells were cultured in normoxic conditions with normal DMEM during the OGD period to be used as the control.

### 3-(4, 5-dimethylthiazol-2-yl)-2,5-di-phenyltetrazolium bromide (MTT) assay

Cell viability was determined by MTT assay. First, we assessed safe concentrations of QDG on HT22 cells. Briefly, cells were seeded into 96-well plates at a density of 10^4^/well for 24 h. After attachment, cells were incubated with QDG (0, 3.125, 6.25, 12.5, 25, 50, 100, 200 µg/mL) for 24 h. Following incubation, 100 µL of MTT-containing solution (0.5 mg/mL in PBS) was added to each well and incubated at 37 °C for 4 h. The resulting purple-blue MTT formazan precipitate was dissolved in 100 µL of DMSO, and absorbance was detected at 570 nm using a plate reader (model ELX800; BioTek, Winooski, VT, USA). To evaluate the protective effect of QDG on HT22 cells injured by ODG/R condition, cells were challenged with OGD conditions for 4 h and then exposed to QDG (0, 6.25, 12.5, 25, 50 µg/mL) for 24 h. Cells cultured under normoxic conditions with normal DMEM during the OGD period were used as the control and MTT assay was performed as described above. The cell viability was determined using the formula: Cell viability (%) = sample optical density (OD) / control OD × 100.

### Hoechst 33258 staining

Cell apoptosis was determined by Hoechst 33258 staining. Briefly, cells were seeded into 6-well plates at a density of 2×10^5^/well for 24 h. After attachment, the cells were divided into 4 groups. Control group: the cells were cultured in normoxic conditions with normal DMEM during the whole period. QDG group: the group was the same as the control group, except that the cells were treated with QDG for 24 h. OGD/R group: the cells were challenged with OGD conditions for 4 h and then returned to normoxic conditions with normal medium for another 24 h. OGD/R+QDG group: the cells were challenged with OGD conditions for 4 h and then returned to normoxic conditions treating with QDG for another 24 h. All cells were fixed using paraformaldehyde for 30 min at room temperature followed by washing twice with PBS. Afterwards, cells were stained with Hoechst 33258 solution for 10 min at room temperature. After being washed twice with PBS, the images of the cells were visualized by a fluorescence-capable microscope (Leica DMI4000B, Germany).

### Experimental animals

Male Sprague-Dawley (SD) rats, weighing 280±20 g, were purchased from SLAC Laboratory Animal Technology Co., Ltd. (Shanghai, China). All rats were housed in specific pathogen-free conditions with controlled humidity, temperature (22 °C), and a 12 h light/dark cycle with free access to food and water. The animal experimental procedures were in adherence to international ethical guidelines and the National Institutes of Health Guide concerning the care and use of laboratory animals, and were approved by Fujian University of Traditional Chinese Medicine Animal Care and Use Committee.

### Model of middle cerebral artery occlusion/reperfusion (MCAO/R)

The rats were administered 2% pentobarbital sodium (0.225 mL/100 g) and restrained on the operation table after anesthesia. The left common carotid artery (CCA), the external carotid artery (ECA), and the internal carotid artery (ICA) were isolated by a short incision. Subsequently, the CCA and ECA were ligated and a silicon-coated filament (0.38 ± 0.02 mm in diameter for rats) was gently inserted from CCA into ICA and advanced approximately 18 mm beyond the carotid bifurcation to occlude the middle carotid artery. After 1 h of occlusion, the filament was removed for about 10 mm from the ICA. Rats were placed on a homeothermic heating pad maintained at 37 ± 0.5 °C during the entire operation to maintain body temperature. Sham-operated rats were subjected to the same surgical procedure without occlusion. The rats were euthanized at the 8^th^ day after MCAO/R surgery.

### Animal group and treatment

Sixty-four modeled rats were divided into 4 groups (n=16 per group): Sham-operated rats were given saline (Sham); MCAO/R rats were given saline (Model); MCAO/R rats were given 0.8 g/kg/day QDG (QDG-L); MCAO/R rats were given 1.6 g/kg/day QDG (QDG-H). Starting from the 1^st^ day after the MCAO/R surgery, rats were administered QDG or saline daily for 7 consecutive days.

### Neurological defect and weight loss assessment

Neurobehavioral deficits were assessed daily after the MCAO/R surgery using a five-point scale: 0, no neurological deficit; 1, failure to completely extend the contralateral fore-limb with a mild neurologic deficit; 2, rotating to the contralateral side with a moderate neurologic deficit; 3, falling to the contralateral side with a severe neurologic deficit; 4, unable to walk spontaneously and unconsciousness. Rats scored between 1 to 3 points were used for further study. The body weight of rats in each group was also measured and recorded daily after the MCAO/R surgery.

### Calculation of infarct volume

The infarct volume was evaluated by magnetic resonance imaging (MRI) at the 1^st^, 3^rd^, and 7^th^ days after MCAO/R surgery. The rats were anesthetized via inhalation of 2% isoflurane and the rate of respiration was monitored to ensure it remaining between 40 and 60/min during the imaging protocol. Rats were placed in a 7.0 T MRI scanner (Bruker, Germany) and immobilized using rat specific coils. The image information of the T2-weighted images (T2WI) phase was collected with a turbo-rapid acquisition relaxation enhancement sequence (Turbo RARE) with the following parameters: repetition time = 4200 milliseconds, echo time = 55 milliseconds, number of averages = 2, field of view = 36 mm × 36 mm, slice thickness = 1 mm, no slice gap, slices = 21, matrix = 256 × 256, flip angle = 90°. The Paravision 6.0 software was used to convert the original images into DICOM format and Image J (National Institutes of Health, Bethesda, Maryland, USA) was used to calculate the infarct volume.

### Cerebral histopathology

Eight rats from each group were anesthetized by 2% pentobarbital sodium and perfused with 0.9% NaCl and 4% paraformaldehyde solution, respectively. Subsequently, their brains were removed quickly. The extracted brain tissues were fixed for paraffin-embedding and cut into a series of adjacent 5-μm thick sections. Sections were deparaffinized and conventionally stained by hematoxylin and eosin (HE). The histopathological changes were assessed and documented by light microscopy (Leica, Wetzlar, Germany).

### TUNEL staining

Sections (5-μm) were collected and prepared as described above, deparaffinized and hydrated, and incubated in proteinase K working solution for 30 min at 37 °C. Following washing with PBS twice, they were blocked for 10 min using 3% H_2_O_2_ at room temperature. Subsequently, the sections were incubated in 50 µL terminal deoxynucleotide transferase (TdT) buffer (45 µL Equilibration buffer + 1 µL FITC-12-dUTP + 4 µL TdT Enzyme) for 1 h at 37 °C. After being washed in PBS 3 times, nuclei were stained with DAPI, and TUNEL positive cells were observed and photographed using a fluorescence-capable microscope (Leica DMI4000B, Wetzlar, Germany).

### IHC analysis

Sections (5-μm) were subjected to antigen retrieval using citric acid buffer, submerged in 3% hydrogen peroxide, washed with PBS 3 times, and then blocked with goat serum for 2 h at room temperature. Sections were incubated with primary antibodies for NeuN (1:1000; Abcam, Cambridge, MA, UK) overnight at 4 °C. Afterwards, the sections were incubated with HRP-labeled secondary antibody (Maixing, Fuzhou, China). Protein expression was visualized using DAB and hematoxylin counterstain, and quantitated by determining the percentage of positive cells in 6 random fields for each sample using a true color multi-functional cell image analysis system (Image-Pro Plus, Media Cybernetics). To exclude any non-specific staining, a second set of sections with PBS substituted for primary antibody was used following the above procedure (negative control).

### RNA extraction and real-time PCR analysis

For *in vivo* experiments, eight rats from each group were sacrificed by administering 2% pentobarbital sodium and the ischemic penumbra of brain tissue from each rat was isolated for RT-qPCR analysis. For *in vitro* experiments, the HT22 cells were divided into 4 groups (as described in the method of Hoechst 33258 staining) and cells were collected for RT-qPCR analysis. Trizol reagent (TaKaRa, Dalian, China) was used to extract RNA from ischemic penumbra tissues and HT22 cells. Subsequently, RNA (l μg) was reverse transcribed into cDNA using Oligo (dT) or special RT-miR-137 primer and PrimeScript RT reagent kit (TaKaRa, Dalian, China) according to the manufacturer's instructions. The RNA levels of lncRNA GAS5 and miR-137 were evaluated using SYBR Premix Ex Taq Ⅱ kit (TaKaRa, Dalian, China) and an ABI 7500 Fast PCR system. GAPDH and U6 were used as internal controls for lncRNA GAS5 and miR-137, respectively. The relative expression of lncRNA GAS5 and miR-137 was calculated using the 2^-ΔΔCt^ calculation method 19.

### Western blot analysis

The protein from the ischemic penumbra of the brain tissues and HT22 cells was extracted using RIPA lysis buffer (Pierce Chemical Co., Rockford, Illinois, USA) containing protease and phosphatase inhibitor. The concentration of protein was calculated using a bicinchoninic acid (BCA) protein assay kit (Pierce Chemical Co., Rockford, Illinois, USA). The extracted total proteins (50 µg) were separated on 12% SDS-PAGE gels and then transferred onto polyvinylidene fluoride (PVDF) membranes (Millipore Corporation, Billerica, MA, USA). Membranes were blocked with 5% non-fat dry milk for 2 h at room temperature, and then incubated with primary antibody against Bcl-2 and cleaved caspase3 (both from Cell Signaling Technology, Inc., Beverly, MA, USA; both diluted 1:1,000); and Bax and β-actin (both from Wuhan Sanying biotechnology Co. Ltd, Hubei, Wuhan, China; Bax diluted 1:2,000, β-actin diluted 1:5,000). All membranes were incubated at 4 °C overnight followed by washing with Tris-buffered saline with Tween 20 (TBST) 3 times. Afterwards, membranes were incubated with corresponding HRP-conjugated secondary antibodies (Wuhan Sanying biotechnology Co. Ltd, Hubei, Wuhan, China; both anti-rabbit and anti-mouse HRP-conjugated secondary antibodies diluted 1:10,000) for 1 h at room temperature. After being washed 3 times, the images of the membranes were visualized by a chemiluminescence reaction (Pierce Chemical Co., Rockford, Illinois, USA).

### Statistical analysis

All the data were analyzed with the SPSS software (version 13.0; SPSS Inc., Chicago, IL, USA). The data are presented as means ± standard deviations. Statistical analysis was conducted by one-way analysis of variance (ANOVA) followed a post hoc test (LSD). A *p* value <0.05 was considered statistically significant.

## Results

### QDG stimulates the growth of HT22 cells after OGD/R

**Figure 1:**
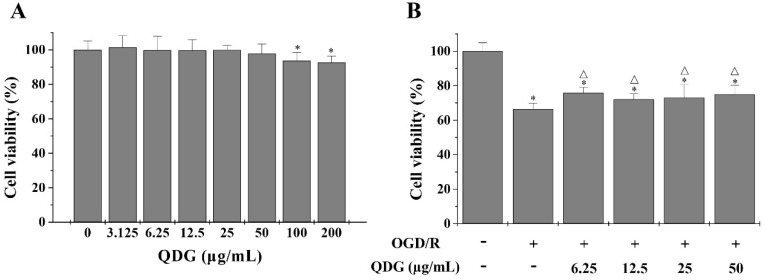
** QDG decreases OGD/R-induced cell damage in HT22 cells.** (A) Cytotoxicity of QDG on HT22 cells. (B) Effects of QDG on OGD/R-induced cell damage in HT22 cells. The cell viability was measured by MTT assay. Data are presented as means ± standard deviations. *p<0.05, compared to control group; Δ*p*<0.05, compared to OGD/R group.

To evaluate the neuroprotective effect of QDG on I/R, we selected mouse hippocampal neuron HT22 cells to establish cell model, which have been commonly used in the study of ischemic stroke *in vitro*. Firstly, cell viability was determined using the MTT assay for evaluating the cytotoxicity of QDG in HT22 cells. Concentrations of QDG with less than 100 µg/mL led to no significant differences in viability compared with untreated cells (Fig. 1A). Furthermore, to determine the protective effect of QDG on OGD/R-induced I/R injury in HT22 cells, we also used the MTT assay. After OGD/R, HT22 cells demonstrated significantly decreased cell viability (66.37±3.48%) compared with the untreated cells. However, treatment with QDG (6.25-50 µg/mL) partly reversed this effect (Fig. 1B). Taken together, these results suggest that QDG has neuroprotective effects on HT22 cells after OGD/R.

### QDG attenuates weight loss and neurological deficits after MCAO/R surgery

**Figure 2:**
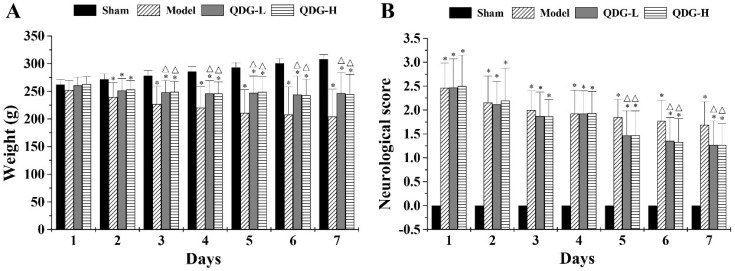
** QDG attenuates weight loss and neurological deficits in rats after MCAO/R surgery.** (A) The weight loss of rats in each group was measured daily. (B) The neurobehavioral score of rats in each group was assessed daily after the MCAO/R surgery using a five-point scale. *p<0.05, compared to sham group; △*p*<0.05, compared to model group.

As weight loss and neurological dysfunction often occur in ischemic stroke progression, we further evaluate the restoration of weight loss and neurological function in MACO/R after QDG treatment. As shown in Fig. 2A, after 2 days post-surgery, the model, QDG-L and QDG-H groups exhibited a significant decrease in body weight compared with the sham group (*p*<0.05), whereas there was no significant difference among the three groups. The 3^rd^ day after surgery, QDG treatment significantly ameliorated the weight loss compared to the model group.

The effect of QDG treatment on the neurological defect in rats with MCAO/R surgery was measured using a five-point scale as described above. The sham group demonstrated no neurological deficits, while the model, QDG-L and QDG-H groups showed substantial neurological deficits (Fig. 2B). However, no significant differences in neurological score among the model, QDG-L, and QDG-H groups were found from the 1^st^ to 4^th^ days after the MCAO/R surgery. Five days post-surgery, QDG treatment significantly improved neurological dysfunction as evidenced by a lower neurobehavioral score compared with the model group (Fig. 2B). Collectively, these results indicate that QDG can ameliorate the weight loss and neurological deficits in rats subjected to MCAO/R.

### QDG decreases infarct volume in rats after MCAO/R surgery

**Figure 3:**
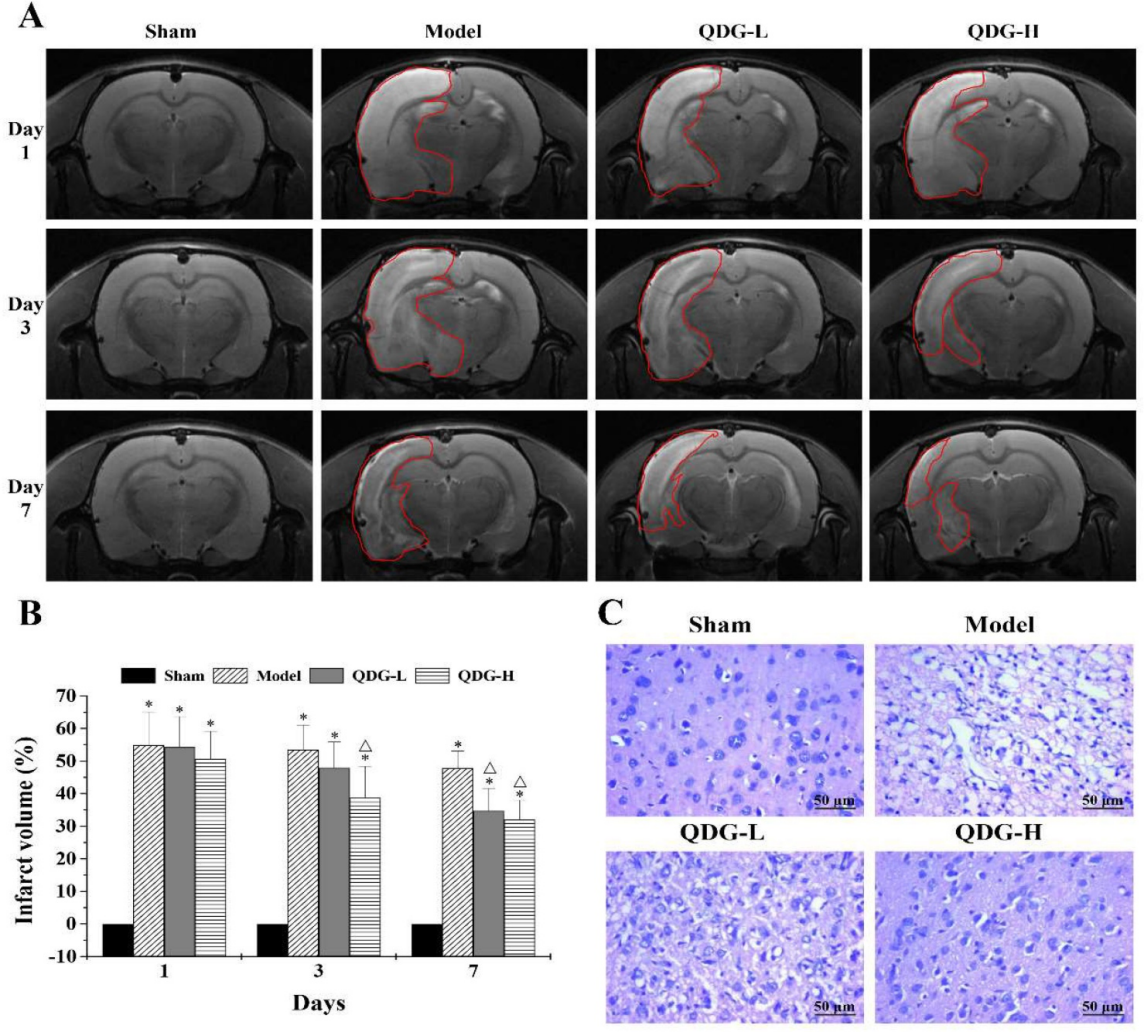
** QDG reduces infarct volume and improves cerebral histopathology in rats after MCAO/R surgery.** (A and B) Representative images of brain infarct volume were determined by MRI scanning at the 1^st^, 3^rd^, and 7^th^ day after MCAO/R surgery and the percentage of infarct volume was analyzed via Image J software. Data are presented as means ± standard deviations of eight rats in each group. *p<0.05 compared to sham group; Δ*p*<0.05, compared to model group. (C) Representative images are shown and were taken at a magnification of 40× by HE staining.

MRI scanning, a safe and non-invasive approach, can dynamically monitor brain infarct volume in the rats induced by I/R. To further assess the protective effect of QDG on the infarct volume after cerebral I/R injury, MRI scanning was carried out. The 1^st^ day after the MCAO/R surgery, there was no significant difference between the model, QDG-L, and QDG-H groups. However, all of these groups showed significantly more severe infarction following the MCAO/R surgery compared with the sham group (Fig. 3A-B), confirming successful establishment of the MCAO/R model. The 3^rd^ day after surgery, the percentage of infarct volume was 53.51±7.54% in the model group, 47.98±7.89% in the QDG-L group, and 38.70±9.67% in the QDG-H group. At the 7^th^ day after the MCAO/R surgery, the percentage of infarct volume was 47.82±5.23% in the model group, 34.75±6.85% in the QDG-L group, and 32.09±5.89% in the QDG-H group. These results suggest that QDG treatment can effectively reduce infarct volume in rats following MCAO/R surgery.

### QDG improves cerebral histopathology in rats after MCAO/R surgery

Ischemic stroke commonly causes severe pathological structural changes in the brain, including cell disorder, nucleolar shrinkage, and breakdown, as well as vacuolization. To demonstrate the protective effect of QDG on brain injury following the MCAO/R surgery in rats, we analyzed changes in cerebral histopathology by HE staining. As shown in Fig. 3C, the cortex area exhibited abundant neurons with normal nuclei and uniform distribution in the sham group. In contrast, the cell morphology of most neurons in the model group showed disorder, shrinkage, nuclear pyknosis, and vacuolization as well as a decrease in cell numbers. Notably, after treatment with QDG, the extent of damage was significantly diminished and the neurons appeared relatively normal. These observations suggest that QDG treatment alleviates pathological changes in rats with MCAO/R surgery.

### QDG attenuates neuronal apoptosis and loss in rats after MCAO/R surgery and in HT22 cells after OGD/R

**Figure 4:**
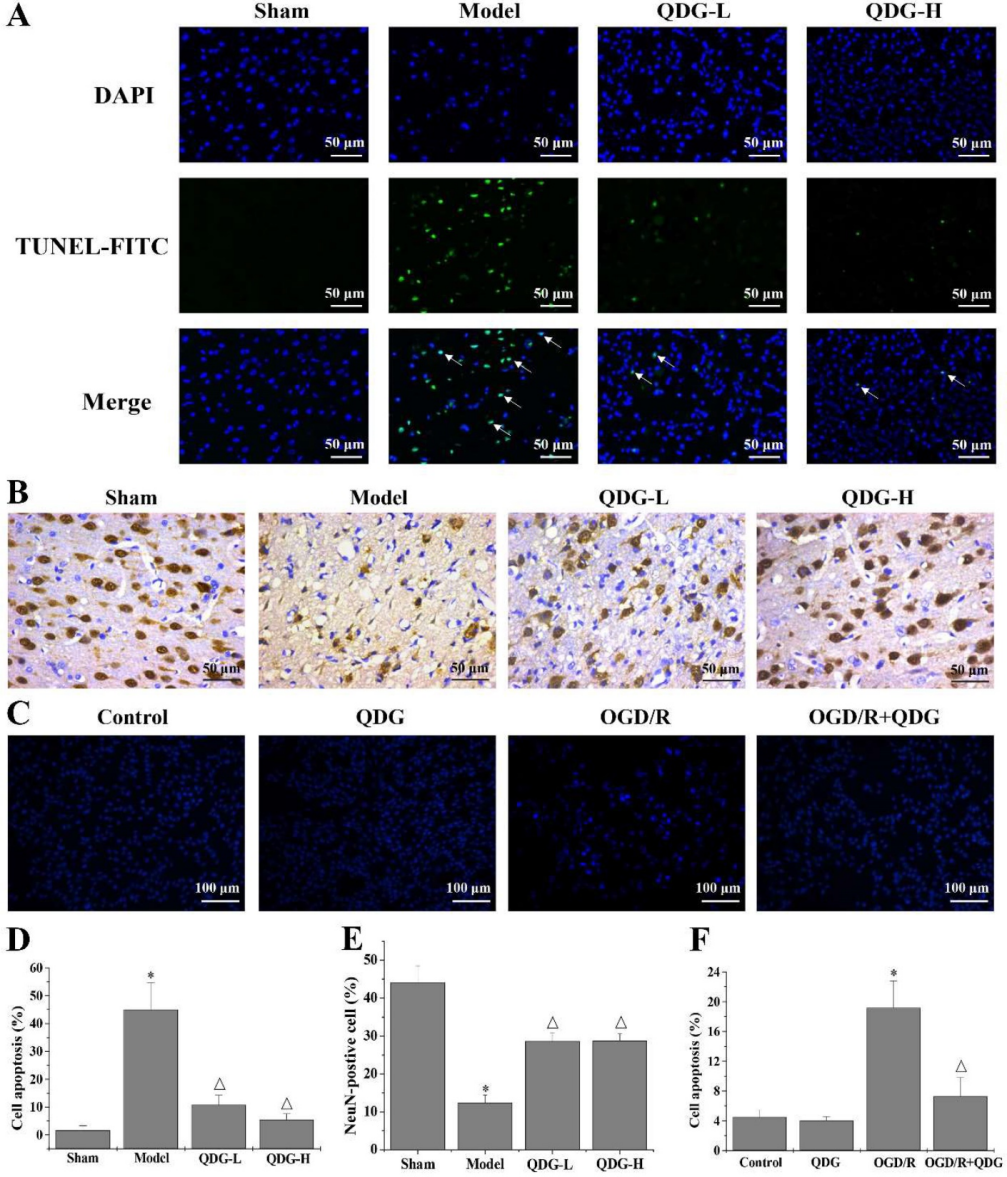
** QDG attenuates neuronal apoptosis and loss in rats after MCAO/R surgery and in HT22 cells after OGD/R.** (A) Representative images of apoptosis in the cerebral cortex area are presented at a magnification of 40× using TUNEL staining. (B) Representative images of NeuN-positive neurons in the cerebral cortex area are presented at a magnification of 40× using IHC analysis. (C) Representative images of HT22 cells apoptosis are presented at a magnification of 20× using Hoechst 33258 staining. (D) The corresponding quantification of apoptosis in the cerebral cortex area was determined by TUNEL staining. (E) The corresponding quantification of NeuN immunoreactivity was determined by IHC analysis. (F) The corresponding quantification of HT22 cells apoptosis was determined by Hoechst 33258 staining. *p<0.05 compared to sham group; Δ*p*<0.05, compared to model group; *p<0.05, compared to control group; Δ*p*<0.05, compared to OGD/R group.

Apoptosis is one of the key pathogenic factors of ischemic stroke. Therefore, TUNEL staining was carried out to detect apoptotic cells in the cortex of the brain. Rats in the model group showed a significant increase in TUNEL-positive cells in the cortex compared with the sham group. In contrast, the number of TUNEL-positive cells in the QDG-L and QDG-H groups significantly decreased compared with the model group (Fig. 4A and D). To further determine if QDG could improve the condition of neurons in the cortex of the brain following MCAO/R injury, IHC analysis was performed to examine the expression of the neuronal marker NeuN. The positive expression rate of NeuN immunostaining was 43.89±4.44% in the sham group, 12.37±2.07% in the model group, 28.61±2.17% in the QDG-L group, and 28.69±1.90% in the QDG-H group, demonstrating that the number of neurons in the brain cortex significantly decreased in the model group compared to the sham group, but significantly increased after QDG treatment (Fig. 4B and E). The condensed nucleus is a typical characteristic of cell apoptosis. To further determine these observations, the Hoechst staining was used to evaluate the effect of QDG on the OGD/R-induced apoptosis in HT22 cells. As shown in Fig. 4C and F, there were no significant differences in the cell apoptosis between the control group and QDG group. Whereas the percentage of apoptotic cells in OGD/R group were remarkably increased, and the cells appeared more nuclear condensation (brightening). However, OGD/R+QDG group effectively elevated the number of living cells and displayed normal. Taken together, these results demonstrate that QDG effectively ameliorated the loss of neurons and apoptosis in the cerebral cortex of rats with MCAO/R surgery as well as OGD/R-induced HT22 cell apoptosis.

### QDG modulates apoptotic-regulatory proteins in rats after MCAO/R surgery and in HT22 cells after OGD/R

**Figure 5:**
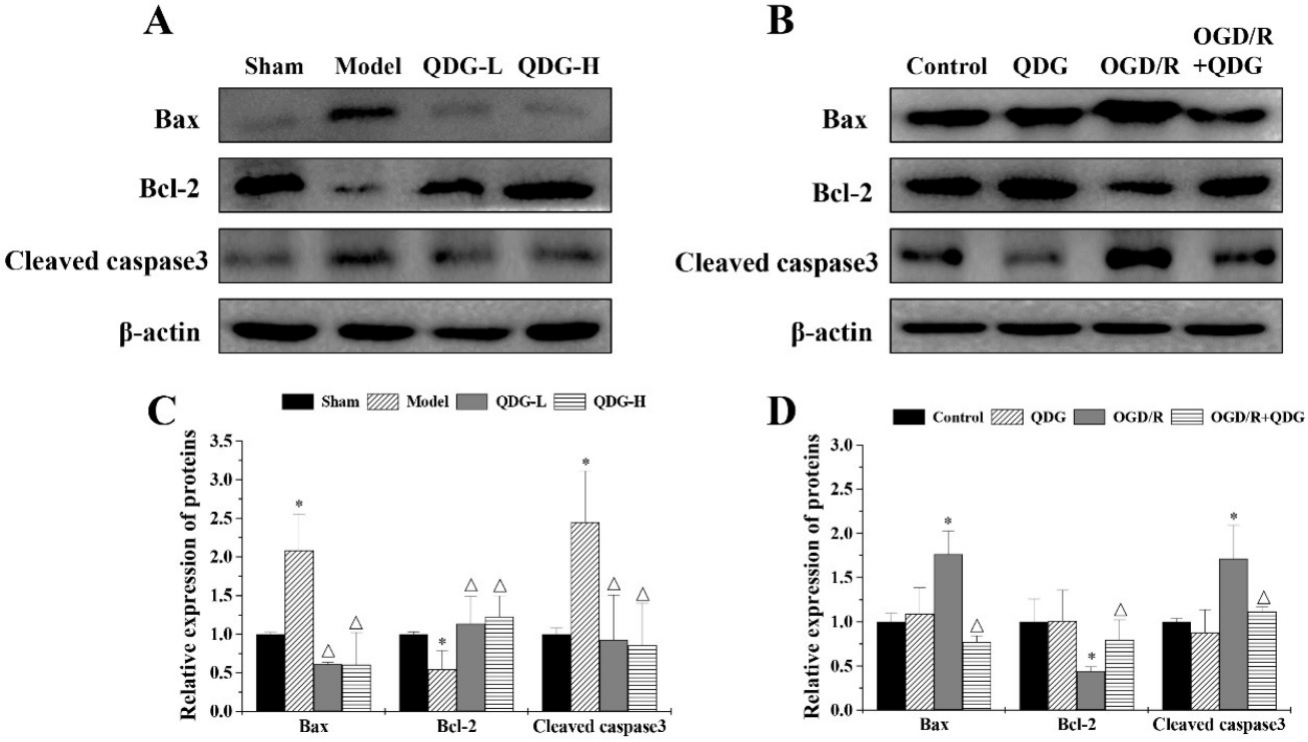
** QDG modulates apoptotic-regulatory proteins in MCAO/R-injured rats and in OGD/R-injured HT22 cells.** (A and B) Protein expression of Bax, Bcl-2, and cleaved caspase3 were determined by western blot analysis. The internal control used was β-actin. (C and D) Relative densitometric findings of the above proteins were determined via Image Lab software. *p<0.05, compared to sham group; △*p*<0.05, compared to model group; *p<0.05, compared to control group; △*p*<0.05, compared to OGD/R group.

To understand whether QDG affects apoptotic-regulatory proteins, we first assessed the expression of Bax, Bcl-2 and cleaved caspase3 in the ischemic penumbra of rats with MCAO/R surgery. There was an increase in the expression of Bax and cleaved caspase3, but a decrease in the expression of Bcl-2 in the model group compared with the sham group, while QDG significantly downregulated the expression of Bax and cleaved caspase3, and upregulated the expression of Bcl-2 compared with model group (Fig. 5A and 5C). To further confirm these findings, we tested the effect of QDG on OGD/R-treated HT22 cells *in vitro*. There were no significant differences in the expression of Bax, Bcl-2 and cleaved caspase3 between the untreated cells and a single treatment with QDG for 24h. However, the expression of Bax and cleaved caspase3 were upregulated and the expression of Bcl-2 was downregulated in the OGD/R group compared with the untreated cells. Conversely, QDG could reduce Bax and cleaved caspase3 levels and elevate Bcl-2 expression in OGD/R-treated HT22 cells (Fig. 5B and 5D). Collectively, these results further confirm that QDG has a protective effect against MCAO/R-induced apoptosis in the ischemic penumbra and OGD/R-induced HT22 cell apoptosis via modulation of apoptotic-regulatory proteins.

### QDG targets the lncRNA GAS5/miR-137 axis in rats after MCAO/R surgery and in HT22 cells after OGD/R

**Figure 6:**
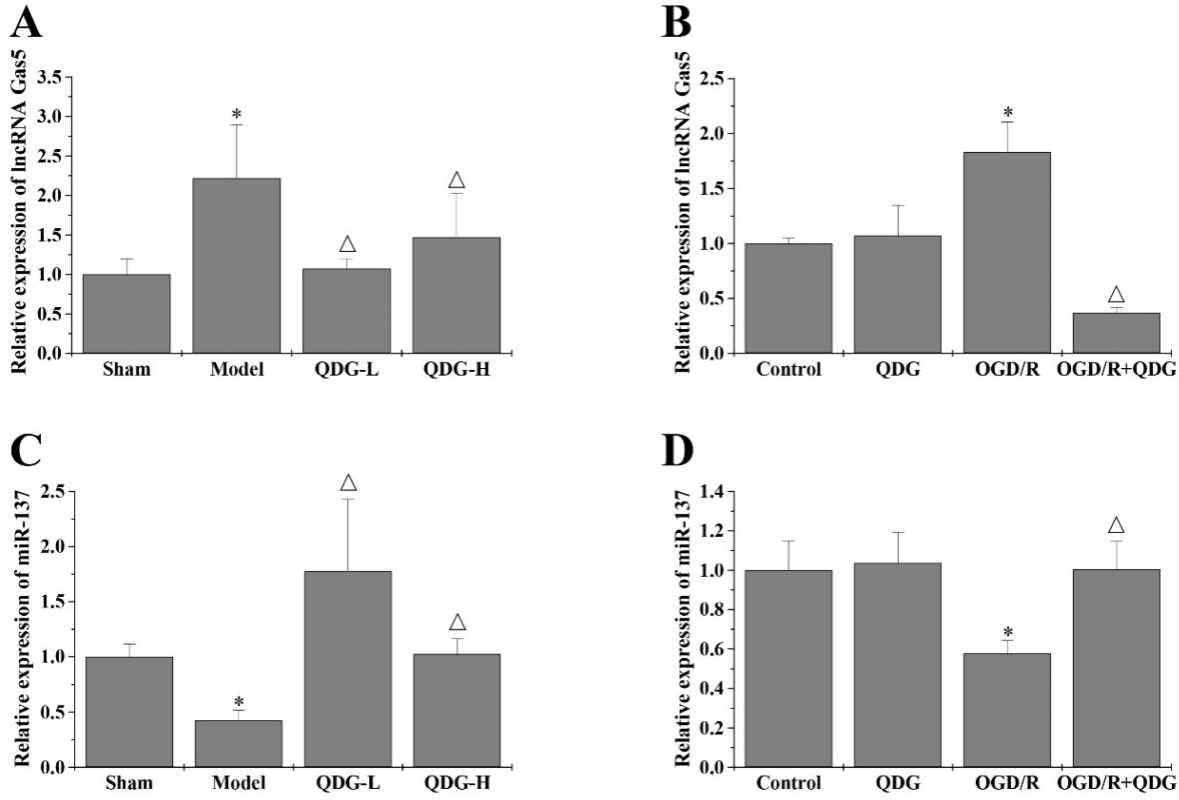
** QDG targets the lncRNA GAS5/miR-137 axis in MCAO/R-injured rats and in OGD/R- injured HT22 cells.** (A and B) The expression of lncRNA GAS5 was determined using RT-qPCR analysis with GAPDH as the internal control. (C and D) The expression of miR-137 was determined using RT-qPCR analysis with U6 as an internal control. *p<0.05 compared to sham group; △*p*<0.05, compared to model group; *p<0.05, compared to control group; △*p*<0.05, compared to OGD/R group.

It has been reported that lncRNA GAS5 can inhibit cell survival during the process of ischemic stroke 15. Previous studies indicate that miR-137 is one of miRNAs competitively bound by lncRNA GAS5 15, which is involved in neuronal apoptosis resulting from ischemic stroke. To evaluate if QDG may ameliorate MCAO/R-induced neuronal death by regulating the lncRNA GAS5/miR-137 axis, we quantified the expression of lncRNA GAS5 and miR-137 in the ischemic penumbra of rats following MCAO/R surgery using RT-qPCR analysis. The model group showed a significant increase in the expression of lncRNA GAS5, but a notable decrease in the expression of miR-137, compared with the sham group. However, QDG treatment significantly decreased lncRNA GAS5 expression and increased miR-137 expression in MCAO/R-injured rats (Fig. 6A and 6C). *In vitro*, there were no significant differences in the expression of lncRNA GAS5 and miR-137 between the control and QDG groups. However, the expression of lncRNA GAS5 was reduced, while miR-137 expression was elevated in OGD/R-injured HT22 cells after incubation with QDG, compared with OGD/R group (Fig. 6B and 6D). These results suggest that QDG inhibits neuronal apoptosis via regulating the lncRNA GAS5/miR-137 axis in rats following MCAO/R surgery and in cells subjected to OGD/R.

## Discussion

Blockage of the cerebral blood vessel causes ischemic stroke, which is the second leading cause of death and disability worldwide 1. Due to the complexity of the central nervous system, neurological deficits are slow to improve. Accumulating evidence suggests that TCM formulations and TCM-derived compounds are effective with few or no side-effects in stroke therapy, which merits further investigation of their underlying mechanisms 20. The TCM formulation QDG consists of 12 g *Gastrodia elata* Blume (Tianma; Rhizoma gastrodiae), 10 g *Uncaria rhynchophylla* (Miz.) Miz. ex Havil. (Gouteng; Ramulus uncariae cum uncis), 6 g *Scutellaria baicalensis* Georgi (Huangqin; Radix scutellariae), and 5 g *Nelumbo nucifera* Gaertn (Lianzixin; Lotus)*.* Numerous studies report that these components play a neuroprotective role in the development of ischemic stroke. 4-hydroxybenzyl alcohol (4-HBA), one of the major active phenolic constituents of *Gastrodia*, has been shown to be an effective agent against transient focal cerebral ischemia via inhibiting cell apoptosis in rats 21. Methanol extract of *Uncaria rhynchophylla* protects hippocampal CA1 neurons against transient forebrain ischemia through its anti-inflammatory action 22. Baicalin, a flavonoid compound extracted from the roots of *Scutellaria baicalensis*, protects neonatal rat brains against hypoxic-ischemic injury by upregulating glutamate transporter 1 via the PI3K/AKT signaling pathway 23. *Nelumbo nucifera* rhizome extracts have been shown to promote cell proliferation and neuroblast differentiation in the hippocampal dentate gyrus in a scopolamine-induced amnesia animal model 24, suggesting that *Nelumbo nucifera* Gaertn may act as a potential neuroprotectant. The powerful neuroprotective effects of these components on ischemic stroke may help to explain why QDG was able to significantly ameliorate I/R-induced cerebral injury in the present study.

Overall, our study suggests that QDG provides neuroprotection against MCAO/R-induced injury *in vivo* as evidenced by reductions in infarct volume in the cerebral cortex and improvements in neurobehavioral deficits in rats with MCAO/R surgery. Notably, we carried out MRI scanning instead of TTC staining to determine the infarct volume. TTC staining is an endpoint procedure requiring sacrifice and processing of animals, which is time-consuming and has the potential to compromise experimental results. In contrast, MRI scanning can continuously monitor brain injury in live animals, allowing dynamic visualization of brain injury progression resulting from I/R. Overall, MRI scanning greatly simplifies the experimental process in comparison with TTC staining and avoids error caused by artificial factors created in TTC staining, increasing the reliability of the results.

Ischemic stroke commonly causes severe pathological structural changes in neural cells, including neuronal apoptosis and necrosis. Some reports suggest that neurogenesis is too limited to allow recovery from ischemic stroke-induced brain injury without timely therapeutic intervention 25. Conversely, improvements in neurogenesis are necessary to regulate neural circuits and improve brain function 26. A large body of evidence indicates that the process of neurogenesis is involved in the proliferation, apoptosis, and migration of neurons 27. In the rats after MCAO/R surgery, the number of neurons decreased and this was accompanied with typical injury characteristics including disorder, shrinkage, nuclear pyknosis, and vacuolization. TUNEL staining results indicated that the neuronal injury present was related to apoptosis. Notably, our results show that QDG can effectively ameliorate neuronal damage in the context of ischemic stroke, as evidenced by an obvious increase in the number of neatly arranged neurons with non-condensed nuclei in the cerebral cortex.

Recently, increasing evidence demonstrates that abnormal expression or dysfunctions in lncRNAs are associated with ischemic stroke 28. Clinically, patients with an insertion/deletion polymorphism (rs145204276) in the lncRNA GAS5 promoter demonstrate upregulated lncRNA GAS5 expression and have an increased risk of ischemic stroke 29. Moreover, downregulation of lncRNA GAS5 *in vivo* inhibits neuronal apoptosis in ischemic brain injury, resulting in decreased infarct volume in the cerebral cortex and improved neurobehavioral deficits 30. Moreover, as downstream target of lncRNA GAS5, miR-137 is also reported with highly expression in the process of neural differentiation, and could regulate neuronal maturation underlying ischemic stroke 31, 32. Consistently, in this study, we also found that the expression of lncRNA GAS5 and miR-137 was inversely correlated in ischemic stroke models. Significantly, after QDG treatment, the inhibition of lncRNA GAS5 expression and the upregulation of miR-137 expression were observed in rats with MCAO/R surgery and in HT22 cells after OGD/R, suggesting that QDG could ameliorate neuronal apoptosis via lncRNA GAS5/miR-137 axis.

## Conclusion

In summary, the present study showed that QDG therapy could reduce infarct volume in the cerebral cortex and improve neurobehavioral deficits in rats with MCAO/R surgery. Moreover, QDG also could inhibit neuronal apoptosis via regulation of the lncRNA GAS5/miR-137 axis. Totally, our findings suggested that QDG may serve as an effective therapy in the clinical management of ischemic stroke.

## Supplementary Material

Supplementary figure S1.

## Abbreviations

CCA: common carotid artery
ceRNA: competing endogenous RNA
DMEM: Dulbecco's modified Eagle's medium
ECA: external carotid artery
HE: hematoxylin-eosin
ICA: internal carotid artery
I/R: ischemia/reperfusion
lncRNAGAS5: lncRNA growth arrest‐specific transcript 5
MCAO/R: middle cerebral artery occlusion/reperfusion
MRI: magnetic resonance imaging
MTT: 3-(4, 5-dimethylthiazol-2-yl)-2,5-di-phenyltetrazolium bromide
OGD/R: oxygen-glucose deprivation/reoxygenation
PBS: phosphate-buffered saline
QXJYD: Qingxuan Jiangya Decoction
QDG: Qingda granule
TBST: Tris-buffered saline with Tween 20
TCM: traditional Chinese medicine
tPA: tissue plasminogen activator
UPLC: Ultra Performance Liquid Chromatography
4-HBA: 4-hydroxybenzyl alcohol
