# Supplementary figures and images for: Qingda granule exerts neuroprotective effects against ischemia/reperfusion-induced cerebral injury via lncRNA GAS5/miR-137 signaling pathway
Source: Int J Med Sci. 2021 Feb 6;18(7):1687–98. doi: 10.7150/ijms.53603 (PMC7976574; doi:10.7150/ijms.53603)

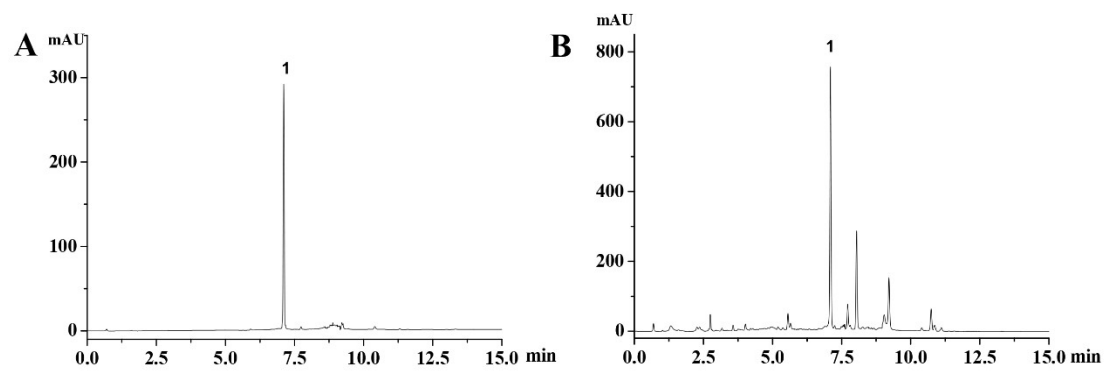

Figure S1. Chromatographic fingerprints of baicalin and QDG by UPLC. (A) 1-baicalin (B) QDG.

Supplement: Supplementary file 1 — Supplementary figure S1. [file ijmsv18p1687s1.pdf]
